# The impact of changing risk thresholds on the number of people in England eligible for urgent investigation for possible cancer: an observational cross-sectional study

**DOI:** 10.1038/s41416-021-01541-4

**Published:** 2021-09-16

**Authors:** Sarah F. Moore, Sarah J. Price, Sarah Chowienczyk, Jennifer Bostock, Willie Hamilton

**Affiliations:** 1 University of Exeter Medical School, College of Medicine & Health, St Luke’s Campus, Magdalen Road, Exeter, UK; 2 Royal Devon and Exeter Hospital, Barrack Road, Exeter, UK; 3 Policy Research Unit in Cancer Awareness, Screening and Early Diagnosis, Queen Mary University of London, Mile End Rd, Bethnal Green, London, UK

**Keywords:** Epidemiology, Health policy, Diagnosis

## Abstract

**Background:**

Expediting cancer diagnosis may be achieved by targeted decreases in referral thresholds to increase numbers of patients referred for urgent investigation.

**Methods:**

Clinical Practice Research Datalink data from England for 150,921 adults aged ≥40 were used to identify participants with features of possible cancer equating to risk thresholds ≥1%, ≥2% or ≥3% for breast, lung, colorectal, oesophago-gastric, pancreatic, renal, bladder, prostatic, ovarian, endometrial and laryngeal cancers.

**Results:**

The mean age of participants was 60 (SD 13) years, with 73,643 males (49%). In 2016, 8576 consultation records contained coded features having a positive predictive value (PPV) of ≥3% for any of the 11 cancers. This equates to a rate of 5682/100,000 patients compared with 4601/100,000 Suspected Cancer NHS referrals for these cancers from April 2016–March 2017. Nine thousands two hundred ninety-one patient-consultation records had coded features equating to a ≥2% PPV, 8% more than met PPV ≥ 3%. Similarly, 19,517 had features with a PPV ≥ 1%, 136% higher than for PPV ≥ 3%.

**Conclusions:**

This study estimated the number of primary-care patients presenting at lower thresholds of cancer risk. The resource implications of liberalising this threshold to 2% are modest and manageable. The details across individual cancer sites should assist planning of English cancer services.

## Introduction

Prompt diagnosis of symptomatic cancer is a priority for patients, the public and governments worldwide. This is particularly so in the UK, which has worse survival from cancer than most comparator countries [1, 2]. In the UK, most patients with symptoms of possible cancer report initially to primary care. In England, selection of patients for specialist investigations for possible cancer is supported by recommendations in the 2015 ‘Suspected cancer: recognition and referral,’ NG12 guideline published by National Institute for Health Care Excellence [3]. This guidance describes presenting features (symptoms, signs and abnormal blood test results) warranting investigation. It established the principle of selecting patients for cancer investigation based on the estimated cancer risk, and chose a threshold of 3% risk warranting urgent investigation. This value was chosen, as a balance between the benefit of liberalised investigation (compared with the previous value of ~5%) and the potential harms of overwhelming clinical services, or over-investigation [3]. Furthermore, for colorectal, lung and ovarian cancers, NG12 supports testing overseen within primary care at levels below 3%, using faecal immunochemical testing (FIT), chest X-ray and Cancer Antigen 125 (Ca125), respectively. Times to diagnosis of patients with the lower-risk presenting features of colorectal cancer have reduced since NG12 was introduced [4].

Efforts continue to improve England’s cancer outcomes, with an explicit target of 75% of new cancers to be diagnosed at stage I or II (i.e. probably curable) by 2028, from the current level of around 54% [2]. One possible intervention to facilitate this would be to lower the threshold risk for urgent investigation to 2%, or even 1%, for all cancers, which could expedite the diagnosis for some patients. Such a change would place further demand for investigations, on top of a considerable recent increase [5]. Demand for lower gastrointestinal endoscopy for all indications doubled from 2012 to 2017 [6]. Imaging figures are similar [7]. Furthermore, there has been unprecedented strain on services during the COVID-19 pandemic, which severely curtailed diagnostic services [8]. Cancer diagnosis is also costly: over £1bn is spent annually in the UK on it [9]. Therefore, any change to the threshold risk for cancer investigations requires estimates of the potential increased demand.

In this study, we estimated how many patients in primary care in England would be eligible for urgent cancer investigations either overseen in primary care or performed in secondary care at a current risk threshold of ≥3%. We extended this to provide estimates for putative ≥1% and ≥2% risk thresholds.

## Methods

### Study design and setting

This was an observational cross-sectional study set in primary care using data from the UK’s Clinical Practice Research Datalink (CPRD GOLD). The study period was 1 January 2015 to 31 December 2016, to allow full capture of participants meeting criteria in 2016. We studied participants’ records for the symptoms, signs and abnormal test results (collectively termed “features” hereafter) for the eleven most common internal cancers: bladder, breast, colon or rectum, endometrium, kidney, larynx, lung, oesophago-gastric, ovary, pancreas and prostate. External cancers that are associated with easily visible signs on clinical examination, such as skin cancers, and haematological cancers, commonly diagnosed through blood tests, were not studied.

### Sample selection criteria

A random sample of 200,000 CPRD participants was supplied by the CPRD, using the following inclusion criteria: aged 40 or over; continuous registration at a CPRD general practice for at least 1 year before 1 January 2016; and complete CPRD records of up-to-date standard throughout the study period. Only participants registered at practices in England were included in our analyses, as there are different diagnostic pathways and guidance across the devolved nations. This left a final study population of 150,921.

### Variables

#### Participant characteristics

We calculated age from the year of birth. Month and day of birth are not available with CPRD data (to protect anonymity) so we assigned a birthday of 1 July. Smoking status adopted the NG12 categories of “never-smoker” and “ever-smoker”.

#### Identification of features

We derived a list of features of cancer from the systematic reviews published in NG12 for each of the cancer sites (Appendix A). For laryngeal cancer, no primary-care evidence was available when NG12 was being drawn up so features representing ≥1% risk of this cancer were identified from a subsequently published study [10].

We assembled CPRD code lists representing each chosen feature, mostly from a previous study [4], supplemented by a small number of new ones, all created using robust methods [11]. These code lists are available in Appendix B.

We searched each participant’s medical records using the code lists, and participant-level variables were created for the presence/absence of each feature. In line with usual practice, we interpreted the absence of a code for a feature as an absence of the feature itself [12].

For abnormal blood test results, we compared a participant’s result with the local laboratory’s reference range. The exceptions to this were tests for anaemia, prostate-specific antigen (PSA) and CA125. For PSA, age-specific values were used: ≥3.0 ng/ml for participants aged 50–69 years and 4.0 ng/ml for ≥70 years [13]. To define anaemia, we used haemoglobin level <130 g/L in men and <120 g/L in women [14]. The threshold for CA125 was 35 U/L, as per NG12.

#### Identification of participants who meet criteria in NG12

We matched each participant’s features with the criteria for urgent investigation in NG12 by cancer site (Appendix A). Appendix A also describes the relevant study or meta-analysis underpinning the selection of features, and identifies the positive predictive value (PPV) of each feature (or combination thereof) for each cancer and the population to which it should apply.

For criteria using paired or recurrent features, we required the second feature to occur within one year of the first, following the methodology in the literature underpinning many of the risk estimates [15].

Within cancer sites, participants were assigned an index date; namely, the date of the earliest time they met any criterion for that site between 1 January 2016 and 31 December 2016. For paired or recurrent features, the index date was the later of the two dates. Participants could have an index date for more than one cancer site.

#### Assignment to risk bands

Within cancer sites, participants were assigned to a risk band (1.0–1.9%, 2.0–2.9%, ≥3%) according to the PPV on the index date. Participants with “Suspected-cancer” codes, but without a coded specific feature, were assumed to meet criteria for urgent investigation under the current NG12 guideline and were added to the ≥3% group. Participants were not assigned to a risk band for any cancers with which they had been diagnosed prior to 1 January 2016. For colorectal, lung and ovarian cancers, triage testing exists within primary care. Test positivity rates in the primary-care setting are estimated as: 15.9% (FIT) [16], 6% (chest X-ray), [Bradley, BJGP, in press] and CA125 (6.8%), respectively [17]. For all three tests, a positive result carries a risk of ≥3% plus a recommendation of specialist referral, so the appropriate percentage was assigned to the ≥3% group.

Within cancer sites, numbers qualifying at each risk band represent numbers of unique participants. The totals across cancer sites comprise multiple contributions from individual participants.

### Analysis

Simple descriptive statistics were used to report the cumulative numbers of participants meeting criteria at the ≥1%, ≥2% and ≥3% risk thresholds within cancer sites. The numbers of participants meeting criteria at each threshold were then converted to a rate per 100,000 and reported with 95% confidence intervals. We estimated the rate (per 100,000 of the population) in England eligible for urgent cancer investigation using totals across all cancer sites for each risk band. These estimates are based on Office for National Statistics data on the English population aged over 40 (27.5 million in 2016; 13.2 million males, and 14.3 million females) [18]. The numbers of actual numbers of 2-week-wait appointments in England for 2016 are reported for comparison [19].

### Study size

There were 1.88 million urgent suspected-cancer appointments in April 2016–March 2017, representing 6.8% of the English population of 27.5 million aged over 40 [18, 20]. A sample of 150,000 gives a margin of error of 0.2 percentage points around an estimate of 6.8%.

### Patient and public involvement

A member of the Patient and Public Involvement (PPI) group affiliated with the Policy Research Unit (PRU) gave feedback on the lay summary of ISAC ethics protocol application to the CPRD for this project and is a co-author on this manuscript. The PRU’s PPI group comprises lay members with an interest as public, patients and/or carers of those with cancer or suspected cancer, and brings the public perspective to our research.

## Results

### Participants

Our sample consisted of 150,921 participants registered at a general practice in England. Participant characteristics are described in Table 1. Comparison to Office for National Statistics UK population data from mid-2016 showed that the sample received was representative in terms of age and sex of the UK population [18].

**Table 1:** Participant demographics.

	Number of participants Number (%)	Age in mid-2016 (years) Mean (SD)
Male	73,643 (48.8)	59.8 (13.6)
Female	77,278 (51.2)	61.5 (14.4)
Total	150,921 (100.0)	60.7 (13.9)

### Participants meeting specific cancer risk thresholds

The features of cancer are included in Appendix A. These are listed by cancer site, identifying criteria for urgent investigation, the positive predictive value (PPV) of each feature (or combination thereof) for that cancer and the population to which they should apply. Appendix A also cites the relevant study or meta-analysis underpinning the selection.

### Outcomes

#### Numbers qualifying for urgent investigation

The cumulative numbers of participants with features of cancer at each threshold of increasing risk are reported as absolute numbers and per 100,000, by cancer site (Table 2). For the three cancer sites (colorectal, lung and ovary) where urgent investigation in primary care is available at the lower threshold of ≥1%, an additional column (under the heading ‘Additional number qualifying for PC testing’) shows the number of participants who would meet these lower threshold criteria. The estimated number of those qualifying for Primary Care testing who would have had a positive test result is shown under ‘Estimated number positive results’. This is added to the numbers meeting the higher-risk criteria of ≥2% or ≥3% to give an adjusted number in our sample and an adjusted number/100,000 population meeting criteria for referral. The percentage change compared to the reference category of PPV ≥ 3% (the current English threshold for urgent investigation) is reported for thresholds ≥2% and ≥1%.

**Table 2:** Number of participants with features of possible cancer in selected risk bands (and comparison to PPV ≥ 3%), in 150,921 English patients aged ≥40 years.

	PPV ≥ 3%							PPV ≥ 2%					PPV ≥ 1%	
	Number qualifying	Additional number qualifying for PC testing	Estimated number positive tests	Adjusted number qualifying for referral after PC testing	Adjusted number/100,000 population after PC testing [95% CI]	Number qualifying	Additional number qualifying for PC testing	Estimated number positive tests	Adjusted number qualifying for referral after PC testing	% change from PPV ≥ 3%	Adjusted number/100,000 population after PC testing [95% CI]	Number qualifying	% change from PPV ≥ 3%	Number/100,000 population [95% CI]
Colorectal	5125	1776	282	5407	3583 [3489, 3678]	5440	1461	232	5672	5	3758 [3663, 3855]	6901	28	4573 [4468, 4679]
Prostate	894			894	592 [554, 632]	1046			1046	17	693 [652, 736]	1126	26	746 [703, 791]
Lung	803	2503	30	833	552 [515, 591]	963	2343	28	991	19	657 [617, 699]	3306	297	2191 [2117, 2266]
Breast	293			293	194 [173, 218]	311			311	6	206 [183, 231]	474	62	314 [286, 344]
Kidney	263			263	174 [154, 197]	263			263	0	174 [154, 197]	961	265	637 [597, 678]
Bladder	246			246	163 [143, 184]	246			246	0	163 [143, 185]	841	242	557 [520, 596]
Upper GI	211			211	140 [122, 160]	249			249	18	165 [145, 187]	5044	2291	3342 [3252, 3434]
Ovary	128	31	2	130	86 [72, 102]	149	10	1	150	15	99 [84, 117]	159	22	105 [90, 123]
Endometrium	109			109	72 [59, 87]	124			124	14	82 [68, 98]	244	124	162 [142, 183]
Larynx	111			111	74 [61, 89]	142			142	28	94 [79, 111]	142	28	94 [79, 111]
Pancreas	78			78	52 [41, 65]	97			97	24	64 [52, 78]	319	309	211 [189, 236]
Totals	8261^a^			8576	5682 [5566, 5800]	9030^b^			9291	8	6156 [6035, 6279]	19517^c^	136	12932 [12763, 13102]

The risk bands are cumulative. Thus a participant in the ≥3% risk band also appears in the ≥2% risk band and the ≥1% risk band.

*Upper GI* upper gastrointestinal (oesophagus or stomach), *PC* primary care.

^a^Total of 8261 in 6898 unique participants.

^b^Total of 9030 in 7453 unique participants.

^c^Total of 19,517 in 10,649 unique participants.

Comparison of estimates of participants eligible for urgent investigation to the actual numbers of patients referred urgently to secondary care in 2016–2017 in England showed a ratio of 1.2 between study estimates and actual numbers of appointments across the cancer sites for which NHS data were reported.

## Discussion

### Key results

This study estimated that people consulted English primary care with features representing a ≥3% risk for one of the 11 commonest cancers at a rate of 5682 (95% CI 5566–5800) per 100,000 adults aged ≥40 in 2016. Liberalisation of criteria for referral from a PPV of ≥3% to ≥2% across the 11 cancer sites would lead to 6156 (95%CI 6035–6279) patients per 100,000 meeting criteria, an 8% increase compared to PPV ≥ 3%. Further reduction to a threshold PPV of ≥1% would have increased numbers eligible for urgent investigation to 12,932 (95% CI 12,763–13,102) patients per 100,000, a 136% increase compared to PPV ≥ 3%.

### Strengths and limitations

This study used CPRD data, which is considered representative of primary care in England, the target population for application of NG12 [12, 21]. Furthermore, our code lists to identify the features of possible cancer were comprehensive and developed by robust published methods: most had been used before [4, 11]. However, there are limitations to CPRD data. Some symptoms of possible cancer will not have been recorded, or were recorded in the ‘free text’ area of the notes, which is unavailable to researchers for fear of compromising anonymity. This missing data will have reduced power, although we had sufficient power to give narrow confidence intervals for our primary analyses. Missing data may also have introduced misclassification bias for symptoms. Analysis of coded data alone underestimates the true number of participants with a symptom, by up to 30% for non-site-specific symptoms such as abdominal pain, and by 20% for symptoms that that are specific and highly predictive of a particular cancer [22]. Thus our estimates of criteria based solely on symptoms, particularly for the 2% and 1% thresholds, are likely to be moderate underestimates. In contrast, test results are automatically coded into the CPRD record, effectively eliminating missing data for that aspect.

We did not attempt to quantify any possible benefits from a liberalisation of the threshold for specialist investigation. The relationship between timeliness of symptomatic cancer diagnosis and improvements in survival has not been studied empirically, although a higher cancer referral rate has been associated with reduced cancer mortality [23]. This relationship is complicated by there being at least three mechanisms whereby survival could be improved: a stage shift, a within-stage shift or an avoidance of complications precipitating emergency admission. One modelling study of the COVID-19-related diagnostic delays estimated a 2-month delay on breast, colorectal and bladder cancers to lead to a reduction in 1-year net survival of 1.4–5.0%, 6.6–10.7% and 9.1–11.6%, respectively, where the ranges represent different age bands from 40 years upwards [8]. If harms from delayed diagnoses were of a similar magnitude to benefits from expedited diagnosis, then an improvement of even 1 week in timeliness of diagnosis could yield a survival benefit, perhaps as much as 1%. Such improvements in timeliness of diagnosis of symptomatic cancer are achievable, as seen following the introduction of NG12 in 2015 [4]. The benefit would not just be experienced by the modest percentage of cancer patients whose sole presenting features were in the 2.0–2.9% range, but also by those who presented with low-risk features before developing higher-risk features. However, we would caution against assuming that an 8% increase in resource use would yield an 8% increase in—for example—improved stage at diagnosis. It may do so—but it may not.

The disparity between the rate of patients eligible for specialist cancer investigation and the rate of actual appointments offered in 2016 was 20%. This is no surprise: it has long been recognised that GPs do not slavishly adhere to guidance, including NG12. Indeed, NG12 explicitly condones this, supporting GPs in making a holistic assessment of the patient’s risk—ideally coming to a shared decision about the need for investigation.

### Use of the results—implications for policy and practice

There are powerful policy implications from this study, particularly relating to any liberalisation of criteria for urgent investigation. Based on our 11 cancers, it appears that a liberalisation to a 2% threshold would have a modest impact on investigative resource use, of around 8%. This increase in resources would not occur evenly across all specialities: in percentage terms, patients with possible pancreatic or laryngeal cancer would see the largest increase.

For colorectal, lung and ovarian cancers, any increase in demand for specialist diagnostic services should be mitigated by primary-care testing. This principle is already present in NG12—explicitly supporting investigation of possible cancer in primary care for risks below 3% where a good primary-care test is available [3]. We incorporated such testing in our methods. However, primary-care testing is not available for all cancers. For upper gastrointestinal cancers there is no current primary-care test that can triage patients with low-risk features, so the additional investigative burden would fall on endoscopy services, already under considerable strain [24]. The resource implications of lowering the investigation threshold to 1% are much larger. In particular, eligibility for possible gastrointestinal cancer testing would increase over 30 fold.

Lowering the threshold also has other implications beyond the scope of this study, in terms of opportunity costs, and potentially increasing patient anxiety and unnecessary investigation.

## Conclusions

These results provide estimates of the impact of changing referral thresholds for suspected cancer on numbers of patients referred across 11 different cancer sites, which can be used to inform future NHS policy in England. The detail of this study across individual cancer sites and criteria will help future planning of cancer services.

## Supplementary information

Appendix A

Appendix B

ISSM STROBE checklist

Reproducibility checklist

## Data Availability

The anonymised participant data from this study are not available, in line with the CPRD’s data security policy. CPRD code libraries are available from the authors on request.
